# MiR-222 Targeted *PUMA* to Improve Sensitization of UM1 Cells to Cisplatin

**DOI:** 10.3390/ijms151222128

**Published:** 2014-12-02

**Authors:** Fangfang Jiang, Wei Zhao, Lijie Zhou, Zifeng Liu, Wenqing Li, Dongsheng Yu

**Affiliations:** Guanghua School of Stomatology, Guangdong Provincial Key Laboratory of Stomatology, Sun Yat-sen University, Guangzhou 510055, China; E-Mails: jiangfangfang0628@163.com (F.J.); zhaowei3@mail.sysu.edu.cn (W.Z.); zhoulj7@mail2.sysu.edu.cn (L.Z.); sumsliu@hotmail.com (Z.L.); wendysums@hotmail.com (W.L.)

**Keywords:** OSCC, PUMA, miR-222, cisplatin

## Abstract

microRNAs have been shown to play critical roles in regulating the chemosensitivity of cancer cells. As a member of the oncogenic miRNAs (oncomiRs), miR-222 has been reported to drive the oncogenesis of many types of malignancies. However, little is known concerning the specific role of miR-222 in human oral squamous cell carcinoma (OSCC). The present study explored the role and mechanism of miR-222 in increasing the expression of p53 up-regulated modulator of apoptosis (PUMA) and enhancing the sensitivity of OSCC to cisplatin (CDDP). Results showed that antisense (As)-miR-222 inhibits the expression of miR-222. In contrast, PUMA was dramaticallyup-regulated. IC_50_ values were significantly decreased in cells treated with As-miR-222 combined with CDDP, to a greater extent than in cells treated with CDDP alone. Furthermore, As-miR-222 enhanced apoptosis and inhibited the invasiveness of UM1 cells. Analysis of the above data suggested that, in UM1 cells, there might be a regulatory loop between miR-222 and PUMA, and that miR-222 inhibition increased the chemosensitivity to CDDP. These findings demonstrated that down-regulation of miR-222 could enhance the chemosensitivity of human OSCC cells to CDDP, and that the combination of As-miR-222 and CDDP could be an effective therapeutic strategy by boosting the expression of PUMA for controlling the growth of OSCC.

## 1. Introduction

Oral squamous cell carcinoma (OSCC) is one of the most common types of head and neck cancer [1,2]. As one of the most useful treatment strategies, chemotherapy plays an important role alongside radiotherapy, surgery, biological therapy, and so on, in the multidisciplinary armamentarium against OSCC. Although the antitumor effects of chemotherapeutic agents have been confirmed in a broad range of malignant tumors, their usefulness is limited due to acquired resistance and severe side-effects [3]. New targeted therapies need to be developed, since the five-year survival rate of OSCC patients remains poor [4,5].

The p53 up-regulated modulator of apoptosis (PUMA), which is induced by p53 tumor suppressors or other apoptotic stimuli, is a newly discovered tumor suppressor and might be a promising new target for gene therapy [6]. It could also influence the chemosensitivity of OSCC cells.

Previous studies have shown that miRNAs can function as tumor suppressor miRNAs or oncogenic miRNAs (oncomiRs) and play important roles in transformation and carcinogenesis [7,8,9]. It was further observed that miRNAs play critical roles in regulating tumor cell responses to chemotherapeutic agents. As a member of oncomiRs, miR-222 has been shown to drive the oncogenesis of several malignancies [10,11,12], and some results have indicated that suppression of miR-222 in human epithelial cancers leads to the repression of cell growth and increasing apoptosis, which could enhance the chemotherapeutic effects of cancer therapy [12]. Analysis of our recent data showed that PUMA was a direct target of miR-222 in OSCC, and that miR-222 could regulate the cell biological behavior of UM1 and Tca8113 by targeting PUMA [13].

As a new strategy, chemical-gene therapy was developed in recent years. It combines traditional chemotherapy with gene therapy and may reduce drug dosages while increasing the efficacy of gene therapy. In the present study, we identified miR-222 as a potent regulator of PUMA and aimed to explore whether suppression of miR-222 could enhance the chemotherapeutic effect of cisplatin (CDDP) on OSCC UM1 cells. The results showed that As-miR-222/CDDP can up-regulate PUMA protein expression and lead to apoptosis in UM1 cells. These findings suggest that PUMA is a novel target of miR-222 and that miR-222 could be a critical therapeutic target for OSCC intervention.

## 2. Results

### 2.1. miR-222 and PUMA Expression in UM1 Cells Treated with Combination Therapy

**Figure 1:**
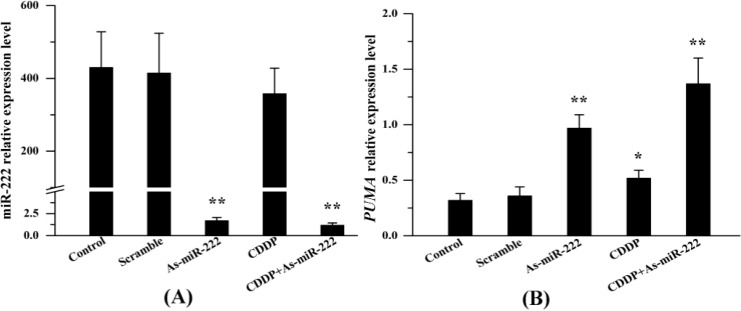
RT-PCR analysis of miR-222 and *PUMA* expression in UM1 cells treated with CDDP and As-miR-222 combination. (**A**) RT-PCR results showed significant down-regulation of miR-222 after transfection with As-miR-222 in UM1 cells; and (**B**) The expression of *PUMA* in CDDP, As-miR-222 and As-miR-222/CDDP groups was significantly up-regulated relative to that in the control and scramble groups. * Denotes statistical significance (*p* < 0.05); ** denotes obvious statistical significance (*p* < 0.01).

As shown in Figure 1, the expression of miR-222 was verified by RT-PCR. Transfection of As-miR-222 altered miR-222 levels significantly compared with the control. It is worth noting that CDDP alone also down-regulated the expression of miR-222, and that the lowest level of miR-222 was achieved by transfection with As-miR-222 in combination with CDDP therapy. For further exploration of the mechanism of miR-222 involvement in UM1 cell biological behavior, the pro-apoptosis gene *PUMA* was measured by RT-PCR. In As-miR-222, CDDP and As-miR-222/CDDP groups, a marked increase of *PUMA* was observed.

### 2.2. As-miR-222 and CDDP Alters Apoptotic Protein Expression

**Figure 2:**
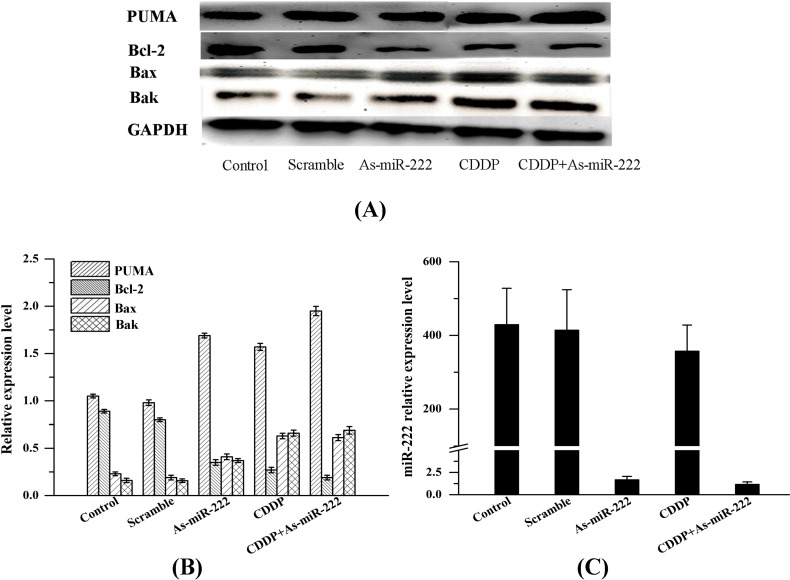
Expression of PUMA, Bcl-2, Bax and Bak in UM1 cells with treatment of As-miR-222 and CDDP. (**A**) As determined by Western blot analysis, PUMA, Bax and Bak were observed to be overexpressed in the CDDP, As-miR-222 and As-miR-222/CDDP groups. In contrast, Bcl-2 exhibited an opposite trend; (**B**) Relative expressions of PUMA, Bcl-2, Bax and Bak were quantified by Image J Instrument software after normalization with the density of GAPDH; and (**C**) miR-222 expression as control detected by RT-PCR.

As-miR-222 and CDDP altered apoptotic protein expression, and the expression of apoptosis-related proteins (PUMA, Bcl-2, Bax and Bak) was measured by Western blot to explore the molecular mechanism of miR-222 involvement in UM1 cell apoptosis. As shown in Figure 2, a significant increase of PUMA was observed in UM1 cells in the CDDP, As-miR-222 and As-miR-222/CDDP groups, especially in the As-miR-222/CDDP group. In contrast, the expression of Bcl-2 in the CDDP, As-miR-222 and As-miR-222/CDDP groups was down-regulated relative to that in the control and mixed groups. Bax and Bak expression was increased as the result of Bcl-2 protein down-regulation. Analysis of the data indicated that As-miR-222 and CDDP could induce UM1 cell apoptosis through activation of PUMA and passivation of Bcl-2.

### 2.3. Determination of PUMA and Bcl-2 Expression in UM1 Cells

**Figure 3:**
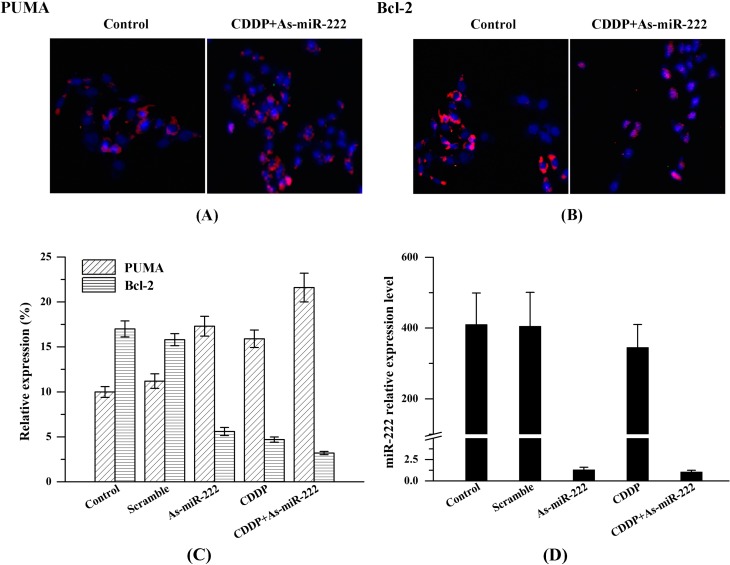
Determination of the expression of PUMA and Bcl-2 in UM1 cells with treatment of As-miR-222 and CDDP by immunofluorescence confocal microscopy; (**A**) Images showed that PUMA was overexpressed with treatment of As-miR-222 and CDDP in UM1 cells; (**B**) The expression of Bcl-2 exhibited an opposite trend compared with PUMA expression; (**C**) Relative expression of PUMA and Bcl-2; (**D**) miR-222 expression as control, detected by RT-PCR.

We performed immunofluorescence staining to determine the expression of PUMA and Bcl-2 in UM1 cells and examined cells using laser scanning confocal microscopy. After immunofluorescence staining, confocal images of UM1 cells showed high red fluorescence of PUMA in the CDDP, As-miR-222 and As-miR-222/CDDP groups; however, the control and mixed groups exhibited relatively low red fluorescence, suggesting weaker expression of PUMA (Figure 3A). In contrast, confocal images showed that the expression of Bcl-2 in the CDDP, As-miR-222 and As-miR-222/CDDP groups was significantly down-regulated compared with that in the control group (Figure 3B). Cell nuclei were stained for blue fluorescence. In various cancers, including OSCC, high expression of Bcl-2 and low expression of PUMA were important characteristics.

### 2.4. As-miR-222 Increases the Cytotoxicity of CDDP on UM1 Cells and Inhibited Cell Proliferation and Invasion

**Figure 4:**
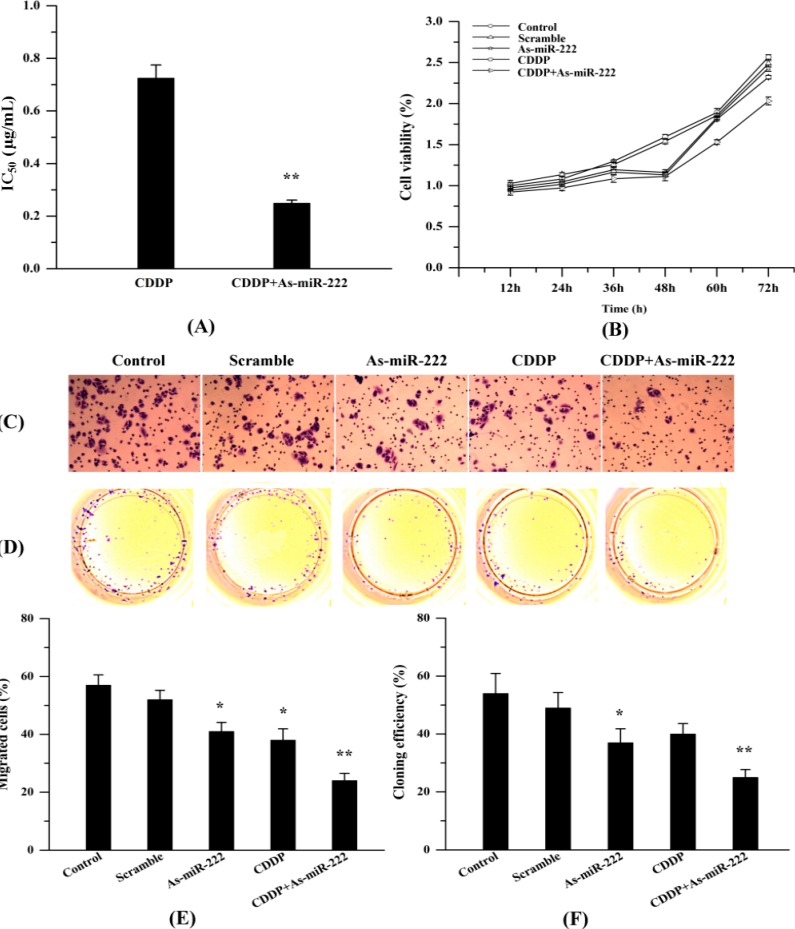
Effect of As-miR-222 on the chemosensitivity of UM1 cells to CDDP treatment and on cell proliferation and invasion. (**A**) IC_50_ of UM1 cells treated with CDDP alone or in combination with As-miR-222; (**B**) MTT assay was performed to detect the growth of UM1 cells treated with As-miR-222 and CDDP; (**C**,**E**) Cell migration was detected by a transwell assay; and (**D**,**F**) Cell-clone-forming experiments were used to detect cell proliferation. * Denotes statistical significance (*p* < 0.05); ** denotes obvious statistical significance (*p* < 0.01).

Dose-response curves were performed for both single CDDP and in combination with As-miR-222. The results suggested that As-miR-222 could increase UM1 cell sensitivity to CDDP treatment and decrease cell proliferation. Figure 4A shows that the CDDP concentration causing 50% growth inhibition (IC_50_) of UM1 cells was 0.725 μg/mL, whereas, in combination with As-miR-222, the IC_50_ was 0.249 μg/mL. Meanwhile, CDDP could also increase the efficacy of As-miR-222. To evaluate the synergistic effect of As-miR-222 with CDDP on cell proliferation and migration, we used MTT assay, transwell and cell-clone-forming experiments to compare the growth of UM1 cells when treated with As-miR-222 alone or with CDDP. As shown in Figure 4, human OSCC UM1 cells treated with As-miR-222 and CDDP proliferated at a significantly lower rate than did other groups, as evaluated by MTT, transwell and cell-clone-forming experiments.

### 2.5. As-miR-222 and CDDP Induced UM1 Cell Apoptosis

**Figure 5:**
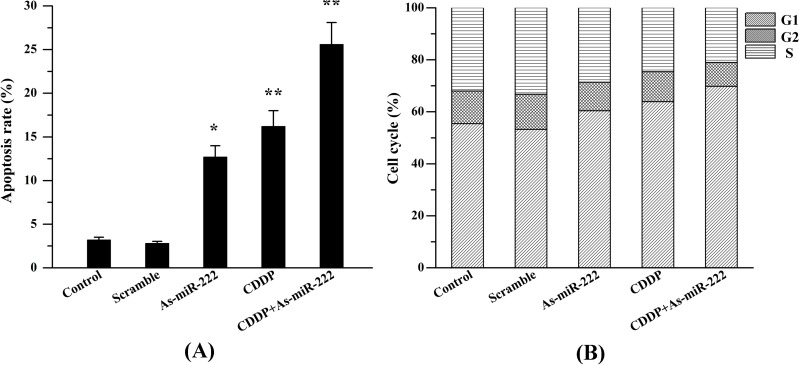
As-miR-222 and CDDP induced UM1 cell apoptosis. (**A**) Percentages of apoptotic UM1 cells are shown in the histogram; (**B**) Flow cytometry analyses were performed to detect UM1 cell cycles in different groups. * Denotes statistical significance (*p* < 0.05); ** denotes obvious statistical significance (*p* < 0.01).

As-miR-222 and CDDP inhibit cancer cell proliferation and induce cancer cell apoptosis. Apoptosis assays were used to test whether As-miR-222 or CDDP inhibited cell proliferation through the induction of cell apoptosis. Annexin V-PI analysis showed that the apoptotic cells were significantly increased in cells treated with As-miR-222 and CDDP together as compared with the other three groups, suggesting that apoptosis was obviously induced in cells when treated with As-miR-222 and CDDP (Figure 5).

### 2.6. miR-222 Acts Directly on PUMA mRNA 3'UTR

**Figure 6:**
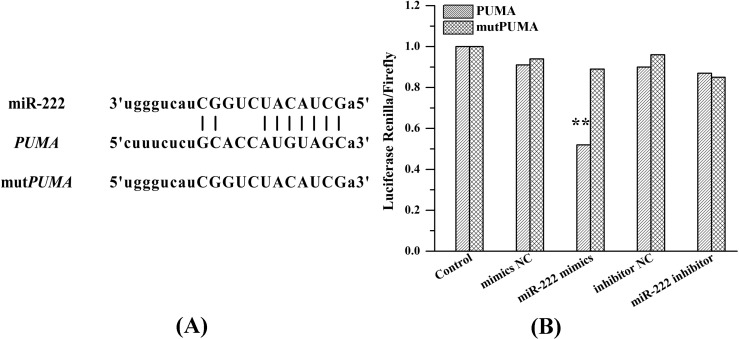
*PUMA* was identified as a target gene of miR-222. (**A**) A panel that described the site of binding between the miR-222 and the *PUMA* 3'UTR and the sequence of *mutPUMA* 3'UTR; and (**B**) Luciferase activity ratio was determined 48 h after 293T cells were transfected with mimics NC, miR-222 mimics, inhibitor NC or miR-222 inhibitor. ** denotes obvious statistical significance (*p* < 0.01).

To validate whether PUMA is a direct target of miR-222, we transfected *PUMA* 3'UTR and *mutPUMA* 3'UTR luciferase constructs into 293T cells with mimics NC, miR-222 mimics, inhibitor NC (negative control) or miR-222 inhibitor. Luciferase activity was measured by means of a dual-luciferase reporter assay system. Figure 6 shows that, compared with other groups, miR-222 mimics increased the activity of 293T cells transfected with *PUMA* 3'UTR.

## 3. Discussion

As a member of the Bcl-2 family, PUMA was discovered in 2001 and was found to possess a powerful pro-apoptotic effect. It belongs to the BH3-only subfamily and is also called Bcl-2 binding component 3 (BBC3). This gene encodes two BH3 domain-containing proteins (PUMA-α and PUMA-β). In contrast to other target spots, PUMA induces apoptosis through not only p53-dependent but also non-p53-dependent pathways to promote apoptosis [14,15,16,17]. Although the specific mechanism for inducing apoptosis needs further investigation, it has been shown to be a promising new target in gene therapy, since the pro-apoptotic function has achieved good results [8,17]. In our study, up-regulation of PUMA and down-regulation of Bcl-2 in UM1 cells were observed with the treatment of As-miR-222 and CDDP. We also found that PUMA 3'UTR mRNA existed in highly conserved miR-222 binding sites and further validated that PUMA was a direct target of miR-222 by luciferase reporter assay. The results suggest that PUMA is directly regulated by miR-222 in OSCC cells.

Previous studies have shown that the expression profiles of miRNAs in chemoresistant human cancer cell lines vary from those of chemoresponsive tumor cells [18,19]. Altered expression of miRNAs could control the chemosensitivity of cancer cells through either directly regulating apoptosis-related Bcl-2 family proteins to modify the cellular response to apoptosis initiated by chemotherapeutic agents, or modulating drug availability to influence the responsiveness of tumor cells indirectly [20,21,22,23]. miRNA expression affected multiple genes simultaneously, and there were some findings providing support for this hypothesis. This was evidenced by the overexpression of miR-221/222, conferring resistance to tamoxifeninMCF-7 cells, and the down-regulation of miR-451 and miR-328, which could result in increased DOX and mitoxantrone sensitivity, respectively [24,25,26]. All the results suggested that the correction of altered expression of miRNAs might have significance for therapeutic strategies to overcome cancer cell resistance. In this study, we chose the UM1 cell line due to its stable performance and demonstrated, for the first time, that the down-regulation of miR-222 expression by As-miR-222 contributed to sensitizing UM1 cells to CDDP. Analysis of our dose-response data showed that As-miR-222 led to an increased sensitivity of UM1 cells to CDDP.

As a member of oncomiRs, miR-222 was frequently up-regulated in various types of human malignancies and has been associated with cell proliferation, invasion, migration and apoptotic ability in a variety of tumors. Several reports have indicated that miR-222 could be used as a therapeutic tool to modulate sensitivity or resistance to anti-cancer agents. Some studies found that MET up-regulated miR-222 expression by targeting *PTEN* and *TIMP3*, enhanced tumorigenicity and conferred resistance to Trail-induced cell death of liver and lung cancer cells [27]. Another study demonstrated that miR-222 could regulate cell invasion, growth and radiosensitivity of gastric cells through directly modulating PTEN expression [28,29,30]. These findings showed that inhibition of miR-222 might form a novel therapeutic strategy, not only to sensitize tumor cells to drug-inducing apoptosis but also to inhibit their proliferation, survival and invasive capabilities.

Current therapy for OSCC needs to seek novel targets and less toxic and more effective therapeutics trategies [31,32,33]. In the present study, the luciferase reporter assay validated that PUMA was a direct target of miR-222.We also found that the combination of As-miR-222 transfection with the treatment of CDDP could significantly inhibit the growth of UM1 cells by reducing proliferation and promoting apoptosis. These results indicated that the modulation of miR-222 activity could regulate the expression of *PUMA* gene and improve sensitization of OSCC cells to CDDP and might represent a novel approach for treating OSCC. These findings also provided new rationales for novel combinational therapies using As-miR-222 to cooperate synergistically with CDDP in patients with OSCC.

## 4. Experimental Section

### 4.1. Cell Culture and Transfection

The UM1 cells were obtained from JCRB (Japanese Collection of Research Bioresources) Cell Bank and maintained in Dulbecco’s modified Eagle’s medium (DMEM) (Gibco BRL, Gaithersburg, MD, USA) supplemented with 10% fetal bovine serum, 2 mM glutamine, 100 μg of penicillin/mL and 100 μg of streptomycin/mL, and incubated in a humidified 5% CO_2_ environment at 37 °C. The 2'-OMe-oligonucleotides were chemically synthesized by Gene Pharma Co., Ltd. (Shanghai, China). The oligonucleotides were modified by 2'-OMe to obtain the following sequences: As-miR-222, 5'-ACCCAGUAGCCAGAUGUAGCU-3'; and scramble sequences, 5'-CAGUACUUUUGUGUAGUACAA-3'. UM1 cells were seeded in 6-well plates and incubated at 37 °C in a humidified 5% CO_2_ environment. Once they were 60% confluent, As-miR-222 and mixed sequences were transfected with Lipofectamine™ RNAiMAX (Invitrogen, Carlsbad, CA, USA) according to the protocol. After 4–6 h, the transfection medium was replaced with fresh medium, and for the combination treatments, cells were incubated with the addition of CDDP. After treatment for two days, cells were divided into five groups, including control, mixed, As-miR-222, CDDP and As-miR-222/CDDP, which were used for further analysis.

### 4.2. RT-PCR

After treatment for 48 h, total miRNAs and mRNA were extracted with the miRNeasy Mini Kit (Invitrogen) and Total RNA Purification Kit (Invitrogen) according to the manufacturer’s instructions. RT-PCR was carried out with the use of a reverse transcription kit with U6, and human β-actin was used as the loading control. For the detection of miR-222 and *PUMA* the SYBR Green PCR assay was conducted, and the primers were as follows: miR-222 (Forward, 5'-ACACTCCAGCTGGGAGCTACATCTGGCTACTG-3'; Reverse, 5'-CTCAACTGGTGTCGTGGA-3'); U6 (Forward, 5'-CTCGCTTCGGCAGCACA-3'; Reverse, 5'-AACGCTTCACGAATTTGCGT-3'); *PUMA* (Forward, 5'-TGTCGAATAAACGCTTTACAAAC-3'; Reverse, 5'-AACGTTTGTAATGATGGCTTCTG-3'); and β-actin (Forward, 5'-GGTCGGAGTCAACGGATTTGGTCG-3'; Reverse, 5'-CCTCCGACGCCTGCTTCACCAC-3'). Relative expression levels were calculated by the 2^−ΔΔ*C*t^ method [34].

### 4.3. Western Blot Analysis

After the treatments for 48 h, UM1 cells were washed three times with cold phosphate-buffered saline (PBS) and subjected to lysis in a buffer composed of 50 mM Tris-HCl, 5 mM EGTA, and 0.1 mM PMSF for the extraction of cellular proteins. Cell lysates were centrifuged, and the concentration of total proteins was determined with the Enhanced BCA Protein Assay Kit (Beyotime Institute of Biotechnology, Haimen, China). The samples were mixed with an equal volume of 5× loading buffer, boiled for 5 min and loaded onto 12% SDS-polyacrylamide gel electrophoresis (SDS-PAGE). After electrophoresis, gels were blotted onto PVDF membrane by electroblotting. The membrane was blocked in TBST (50 mM Tris-HCl, pH 7.5, 0.2% Tween, 150 mM NaCl) containing 5% non-fat milk for 1 h at room temperature. The blots were then incubated with specific primary antibodies against PUMA, Bcl-2 and GAPDH overnight at 4 °C, followed by secondary IgG antibody for 1 h at room temperature and washed three times with TBST. The membrane was developed with enhanced chemiluminescence (ECL) reagents and visualized in the AlphaView SA system (Zeiss, Dresden, Germany) for 1–15 min. Image J Instrument software (Yihui, Guangdong, China) was used to quantify the bands of specific proteins after normalization with the density of GAPDH.

### 4.4. Immunofluorescence

UM1 cells were seeded in 24-well plates with 12-mm-diameter cover slips and incubated for 24 h. Cells were then treated with As-miR-222 or CDDP for the indicated time. After 48 h, cells were washed three times with PBS (pH 7.4), fixed in 4% formaldehyde, permeabilized in 0.1% Triton-X-100 and sequentially incubated with the indicated primary antibodies according to manufacturer’s instructions for 18 h. Subsequently, cells were incubated for 1 h with each of the corresponding secondary antibodies, and nuclei were stained with DAPI.

### 4.5. Cell Viability Assay

UM1 cell viability was evaluated by the 3-(4,5-dimethylthiazole)-2,5-diphenyltetrazolium bromide (MTT) assay. Four thousand cells/well were seeded into 96-well plates and then allowed to attach overnight. After treatment as described above, a 20 μL quantity of MTT (5 mg/mL) was added to each well at 12, 24, 36, 48, 60 and 72 h, and the cells were incubated for 4 h at 37 °C. The reaction was stopped by the addition of 100 μL of DMSO to each well to dissolve the precipitate for 15 min. Optical density (OD) was measured at a wavelength of 570 nm by spectrophotometric analysis. The data were derived from quintuplicate samples of at least three independent experiments.

### 4.6. Cell Migration Assay

UM1 cells (1 × 10^4^) were transferred to 8-μm-pore inserts after treatment with As-miR-222 and CDDP, and then placed in wells that contained medium with 10% FBS. The non-migrating UM1 cells on the upper surface were harvested after incubation for 12 h, and cells on the lower surface were fixed and counted by microscopy.

### 4.7. Flow Cytometry

For cell-cycle analysis, treated and control UM1 cells in the log phase of growth were harvested, washed twice in PBS and fixed in 75% alcohol at 4 °C for 24 h. The nuclei of cells were stained with 50 μg/mL propidium iodide (PI) for an additional 40 min at room temperature in the dark. Cells were re-suspended, and cell cycles of different groups were examined by flow cytometry. Results were presented as the percentage of cells in a particular phase.

For apoptosis assay, Annexin V-PI staining was performed and apoptosis was evaluated by flow cytometry. After treatment with As-miR-222 and CDDP, both attached and floating cells were collected and washed with cold PBS and then re-suspended in buffer at a concentration of 10^6^/mL. According to the manufacturer’s protocol, cells were subjected to Annexin V-PI staining and were analyzed by flow cytometry after 15 min incubation in the dark at room temperature.

### 4.8. Luciferase Assay

The 293T cells which obtained from American Type Culture Collection (Rockvill, MD, USA) were co-transfected with pMIR vectors containing *PUMA* 3'UTR or mut*PUMA* 3'UTR and miR-222 mimics. Cells were subjected to lysis, and the lysate was detected with a dual luciferase reporter assay kit according to the manufacturer’s protocol, with a luminometer. Renilla luciferase activity was used for normalization, and firefly luciferase activity was detected. 

### 4.9. Statistical Analysis

Data were presented as means ± standard deviation (SD) of at least three separate experiments. Results were performed with SPSS software 17.0 (SPSS, Shanghai, China) by *t*-test or one-way analysis of variance (ANOVA). A *p*-value less than 0.05 was considered to be significant.

## 5. Conclusions

Our results indicated that the down-regulation of miR-222 could improve the sensitization of oral squamous cell carcinoma UM1 cells to CDDP by boosting the expression of the *PUMA* gene. These findings suggested that a combination of As-miR-222 and CDDP could be an effective therapeutic strategy for the treatment of OSCC.
